# The Changing Medical Curriculum

**Published:** 1969-04

**Authors:** 


### THE CHANGING MEDICAL CURRICULUM

A large gathering of students, practising doctors, and some medical teachers attended a " Teach-in " on Medical Education at the Medical School on 15th January. This very successful event was organised jointly by the medical students' society, Galenicals, and the Bristol Division of the British Medical Association, whose chairman, Dr. K. C. P. Smith, presided. In introducing the principal speaker, Sir Ronald Tunbridge, Professor of Medicine at Leeds University and Chairman of the B.M.A. Board of Science and Education, Dr. Smith referred to the recent " mini-explosion " of Government papers (Todd, Seebohm *et al.*) which had aroused considerable interest in the medical curriculum and medical education in general.

*Professor Tunbridge* reminded the audience that interest in medical education is fairly recent going back only some 240-250 years, a standardised course of training having been forced on the profession by the increasingly numerous affluent patients who had demanded something better for their money than the haphazardly trained physicians, surgeons, and apothecaries of earlier centuries. Three factors controlling the training of a doctor were, he said, the scientific knowledge of the period, the climate of opinion in the community at the time, and the system of organisation in the area where he would practise. One often heard it said that the aim should be to produce "a good G..P." but at present only some 40% of medical graduates entered general practice; another 40% took up hospital work, the remaining 20% being variously employed in teaching, public health, research, etc. The trends were such that in 10-15 years time the figures might well be 30%, 60% and 10%. No longer was a doctor on qualification regarded as fit to practise medicine anywhere and it must be accepted that some form of specialist training was necessary for every graduate; in this context, he would regard general practice as a specialty in its own right.

Discontent within the profession, said Professor Tunbridge, was not concerned so much with finance as with status. Until recently the doctor in a small community was, like the parson and the schoolmaster, one of the few people who had had a university education. In these days when some 10% of the population attended universities or colleges, the doctor no longer had this privilege and was not automatically a leader of society. He would only retain his position if he was good at his job and the aim of medical education must be to produce first-class professional people. Moreover in spite of the constant impact of new knowledge and the fact that perhaps 80% of what is being taught today will be out of date in 20 years time he must keep up to date; a first class professional person could not afford to sit back when older and rest on what he had been.

Another myth was that medical training must be based on apprenticeship. He would not like to see this system disappear completely but we must rethink the role of apprenticeship in modern training. Newer methods of learning could shorten the time taken to learn and these should be exploited wherever possible. Doctors, traditionally were individualists but there is no longer much that the doctor can do alone. The team approach must be cultivated; the taking of one decision on one patient could involve over 200 people, many of them non-medical. Medicine was no longer a charity. The public expected the best and we should do our best to provide what the community needed.

*Mr. N. Temple,* president of Galenicals, referred to the activities of the Medical Curriculum Reviewing Committee. It seemed that present intentions were that the pre-clinical course would extend over two years embracing anatomy, physiology, biochemistry, cell biology and behavioural science. The first two clinical years would be spent in clinical appointments with two afternoons a week throughout the period being devoted to an "integrated course" in para-clinical subjects with participation of clinical specialists as necessary. This would be followed by the final written examinations in surgery, medicine and para-clinical subjects. The final clinical year would be an "elective" year consisting of a period of special study or an assistant house appointment or a period of revision or a combination of the above. At the end of this year there would be practical examinations with consideration of any dissertation arising from a period of special study. There would also be a system of continuous assessment of the student's progress throughout the clinical years. It seemed to Mr. Temple that the most valuable part of these proposals was the course of integrated studies. He appeared undeterred by the thought of 240 students being taught at a time for a period of several hours on two afternoons every week.

Another factor leading to changes in the curriculum was the increasing number of students. This must lead to re-organisation of courses and the use of beds in Regional Board hospitals for teaching. The first few students to experience the latter arrangement at Southmead Hospital had reported that the staff were fresh and that the teaching was different and exciting, when compared with some other institutions. He emphasised the active interest which students were taking in the curriculum, the amount of talking and discussion that went on and the desire to have a hand in planning. Students might be inexperienced and immature but, as consumers, they were entitled to have their say.

He would like to see more teaching by general practitioners. It was difficult for hospital specialists to talk about their specialty as if it were as unimportant as it really was. G.P.'s could do this and if students were not to lose their humanity and become scientific technologists they needed more opportunity to see medicine in the community.

*Dr. E. Williams,* a past chairman of the Junior Doctors' Association, dwelt on economics. Undergraduate medical education at present costs £30-35 million per annum. If the Todd report proposals were implemented it might cost £80 million in 1990. The present shortage of doctors could be wiped out by 1985 by doubling the number of students. The proportion of the gross national product spent on health in the U.K. was 4.8%. In U.S.A., Scandinavia and Germany it was more than 6% and even in France it was 6.3%. It did seem therefore that our Welfare State was somewhat parsimonious and Dr. Williams could not see the necessary funds forthcoming. We should therefore have to produce cheaper doctors. It had been suggested that this could be done by less teaching, better use of teaching buildings, giving students loans instead of grants and by encouraging students to live at home. Why not go all the way and organise dawn-to-dusk dissecting schedules, midnight rounds and broadcast review articles at meal times? Why not indeed?

Turning to post-graduate education, it seemed that unless a doctor emigrated or became an anæsthetist a period of about ten years post-graduate study had to be grafted on to five years under-graduate training. Even Todd envisaged one year's internship, three years general professional and three years special professional training. To achieve this it would be necessary to use all available hospitals and all available teaching facilities. It was odd that the Todd report made no mention of the teaching functions of non-consultant hospital staff. Since there were only 420 full-time clinical teachers in the whole country these part-time teachers must do an awful lot of teaching and should therefore be given time to teach.

*Dr. Ian Olson,* a pre-clinical teacher, stressed that pre-clinical departments not only had to resolve conflicts between teaching and research but also between demands for vocational as distinct from purely scientific teaching. With less than half the pre-clinical teachers in Bristol being medically qualified the tendency was for the teaching to be scientific. The introduction of a B.Sc. degree which would be taken before proceeding to clinical studies had been suggested but he felt that to break down the barrier between pre-clinical and clinical curricula might be better. It could be that it was the present structure of the University with its rigid division into faculties and departments which stood in the way of this. After all if the University were to appoint a Professor of Witchcraft who would in turn appoint three lecturers and three assistant lecturers in Witchcraft, it would become difficult to keep witchcraft out of the curriculum. Instead he commended the interdepartmental approach; decide what should be taught and then select men from a variety of departments to do it.

*Dr. Michael Lennard,* a general practitioner, asked the question: What is vocational training? It really is, he said, what all doctors have been getting for themselves for the last 40 years. They had to get it as best they could; now an attempt was being made to systematise it. There should be a kind of registrarship in general practice but the difficulty would be to find enough posts, enough teachers and enough incentives. On graduation a student should have become a "well-educated, totipotential, free-swimming, fœtal physician". He should be aware of the potential of each branch of medicine. Just as he has learnt the potential of surgery in the "workshop" of the out-patient department and operating theatre, so he should learn about medicine in the community in the "workshop" of the health centre, the clinic and the family practice. He could not be taught it in hospital practice since this would insensibly condition the student against medicine outside the hospital.

*Mr. Richard Harding,* a student who had achieved sixth year status, was not entirely satisfied by what he had gone through. He felt that there was too much specialisation and not enough about medicine in the community. It was difficult to discuss with teachers the general effects of medicine on the community, e.g. why prolong life in certain cases?, population problems and so on. The two weeks spent with a general practitioner had been very helpful; he seemed to have more time to talk about these problems. Could students not have a loose attachment to a G.P. throughout their course?

The proposal for an elective period in the sixth year seemed to him a good one. He felt that pre-clinical students needed more information as to the relevance of the mass of science they were being taught. The attempt to integrate subjects as in the cell biology course was to be commended.

A lively and wide-ranging discussion ensued. Several students complained of too much specialisation and a too rigid distinction between pre-clinical and clinical courses. These was a request for an earlier introduction to hospital work and even a revolutionary suggestion that anatomy and physiology might come later in the course. A physician maintained that anyone who had applied for such a post knew that there was no lack of applicants for full time clinical teaching appointments; why not use this reserve of talent for pre-clinical teaching? A member of the Public Health Department found it difficult to visualise integrated teaching for 240 students at a time. He asked if specialisation necessarily meant compartmentalisation and suggested that informal arrangements for interfertilisation might be the answer. Another speaker complained of a general lack of understanding of what is meant by teaching and education. Many students with a scientific training could not, he said, reason logically, many senior doctors learnt more slowly than when they were students and few teachers know how to transfer knowledge.

Saying that students did not ask for nearly enough, the Clinical Dean at Southmead Hospital recalled a recent discussion between the pre-clinical professors and the hospital staff. It was interesting that what the Regional Hospital Board consultants had said on that occasion was almost the same as the students were saying now. He hoped that professional teachers present would take notice of this. Unlike Professor Tunbridge he believed that, in the future, medicine in the community would become more, not less, important.

A student enquired how much students or those in close touch with them had a part in designing the new curriculum. She felt that bad " firms " did not ask how their teaching could be improved; the good ones did, but they were good already. She felt that there should be one single person in overall charge of undergraduate medical teaching.

In reply to more student requests for participation in planning the new curriculum, the Dean said that student consultation was welcomed and that the mechanism for this had been established.

Several general practitioners commended the suggestions that had been made for continuous contact with students throughout the clinical course.

Summing up, *Professor Tunbridge* said that he could not help being old-fashioned. If one read old copies of the *Lancet* one would find that much of what had been said tonight was said every ten years or so. It was quite right that students should have views on the effectiveness of their teachers but they could not know which way medicine was going. Teachers could only hope to get over a broad general basis on which the student himself would have to build.

Clinical work from the beginning of the course had now been abandoned, even in France. Integrated teaching had been pioneered in Western Reserve University where the staff had gone into retreat for two years to prepare the course; it had proved difficult to maintain continuity when staff left and had to be replaced; similar difficulties were being experienced in Newcastle. It should not be forgotten that integration also occurs in the student's mind as part of the process of learning. Another problem was that a different approach was needed for students at various stages of their career. He regarded the elective year as very important, giving the student an opportunity to study something in depth or to travel.

It was right that much thought and discussion should go into planning the curriculum but we should not lose sight of what we were trying to produce—men and women of culture and education, who in this framework would practise our profession.

### REFERENCES

GREAT BRITAIN; Royal Commission on Medical Education.

Royal Commission on Medical Education, 1965-68; report 1968 Cmnd. 3569 (" Todd Report ").

GREAT BRITAIN; Parliament.

Report of the Committee on Local Authority and Allied Personal Social Services 1968. Cmnd. 3703 (" Seebohm Report ").

